# Carotid artery stiffness induced by the fine particulate matter PM2.5 could be alleviated by exercise

**DOI:** 10.1111/cns.14488

**Published:** 2023-10-07

**Authors:** Ying Jiang, Hai‐Bo Cao, Qin‐Qin Shu, Zheng Xu, Li‐Ling Wang, Yan‐Jun Guan, Jie‐Qing Wan

**Affiliations:** ^1^ Cerebrovascular Diseases Center, Department of Neurosurgery Renji Hospital, Shanghai Jiao‐tong University School of Medicine Shanghai China; ^2^ Department of Neurosurgery Suzhou Xiangcheng People's Hospital Suzhou China; ^3^ Department of Emergency Medicine Shanghai No. 4 People's Hospital affiliated to Shanghai Tongji University School of Medicine Shanghai China; ^4^ Department of Otorhinolaryngology Shanghai Rui‐Jin Hospital, Shanghai Jiao‐tong University School of Medicine Shanghai China

The ultrafine particle matter PM2.5 (diameter size <2.5 μm) has attracted the most attention due to its significant impact on human health. PM2.5 can not only cause direct damage to the entire respiratory system, but also penetrate the respiratory epithelium and enter the blood circulation,^1^ which eventually damages all organs inside the body. Recently, a tight correlation has been suggested between PM2.5 and stroke.^2^ However, most of these studies stayed at the epidemiological level and the detailed mechanisms remain unknown.

The primary cause of stroke is cerebral and carotid artery lesions.^3^ Whether it is because of physiological aging or pathological disease, cerebral/carotid artery lesions will all appear as a series of pathological changes, which will eventually lead to decreased arterial elasticity, a.k.a. atherosclerosis. During clinical practice, the pulse wave velocity (PWV) acquired by Doppler can directly reflect the arterial stiffness and hence has been used as the gold standard for measuring the severity of atherosclerosis. However, it remains inconclusive whether PM2.5 could cause cerebral/carotid arterial lesions.

In this study, we characterized the pathological effect of PM2.5 on promoting carotid artery stiffness via damaging endothelial cells. Since the brain‐derived neurotrophic factor (BDNF) is highly expressed in endothelial cells and has been demonstrated to protect endothelium,^4^ we further demonstrated the counteractive effect of BDNF on the toxicity of PM2.5. The data normality was checked by the Shapiro–Wilk test.

We first investigated the changes in carotid artery PWV after different PM2.5 intraperitoneal dosages. Simple main effects analysis showed that PM2.5 had a statistically significant impact on carotid artery stiffness [*F* (5, 240) = 6.7, *p* < 0.001]. By the end of dosing (Week 2), there remained no difference in PWV among all groups (*p* > 0.05). This trend persisted until Week 18, when a dramatic increase in PWV was observed in the PM2.5 dosed at 1000 ng/g as compared to the control group (*p* < 0.05). This difference was further widened in Weeks 22 (*p* < 0.01) and 26 (*p* < 0.05) (Figure 1A).

**FIGURE 1:**
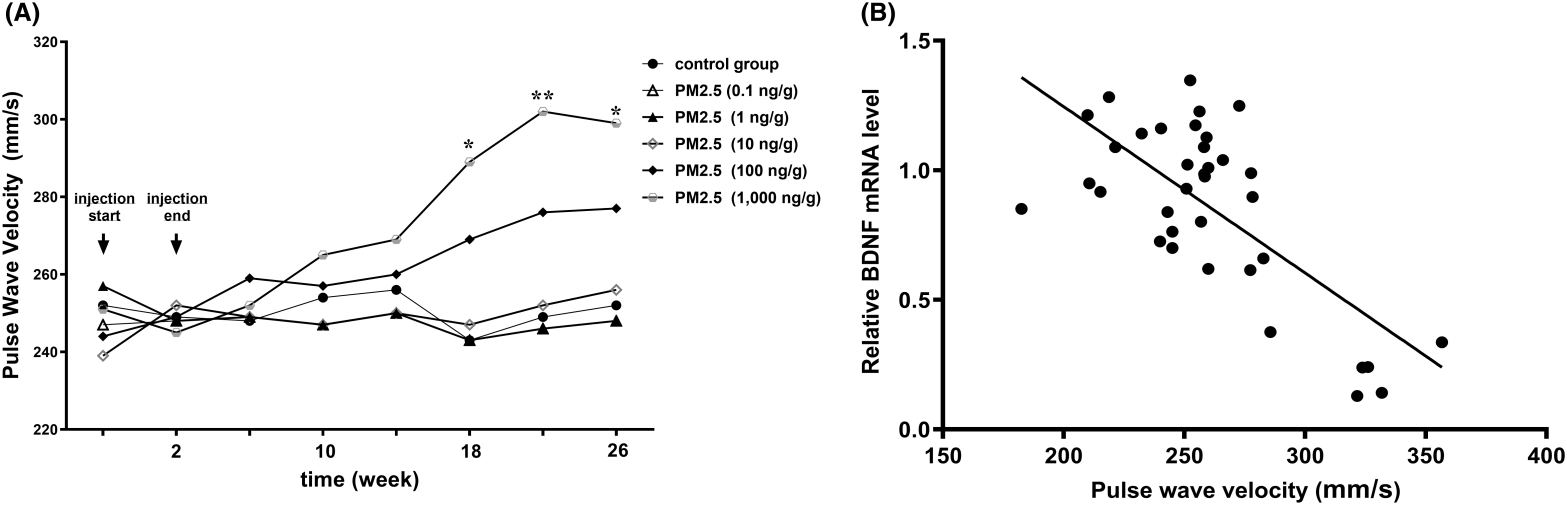
The toxic effect of PM2.5 on the carotid artery. (A) Since Week 18, a dramatic increase in PWV was observed in the PM2.5 group (1000 ng/g) as compared to the control group (*p* < 0.05). This increase persisted in Weeks 22 (*p* < 0.01) and 26 (*p* < 0.05). (B) A negative correlation was observed between the endothelial BDNF mRNA level and pulse wave velocity (PWV) of the carotid artery (*R* ^2^ = 0.5, *p* < 0.001).

We next collected the endothelial cells of the carotid artery on Week 26. qPCR demonstrated a prominently decreased BDNF mRNA level in PM2.5 1000 ng/g group as compared to that of the control (*p* < 0.01). A negative correlation was observed between the endothelial BDNF mRNA and PWV (*R* ^2^ = 0.5, *p* < 0.001) (Figure 1B).

We then tried to elevate animals' BDNF concentration via treadmill exercise as previously described.^5^ On Week 26, both exercise [*F* (1, 28) = 28.76, *p* < 0.01] and PM2.5 [*F* (1, 28) = 36.14, *p* < 0.01] demonstrated significant impacts on BDNF expression inside the endothelial cells of the carotid artery (Figure 2A). Post hoc analysis indicated that exercise could dramatically promote BDNF expression in the circumstance of PM2.5 dosing (*p* < 0.01), which alleviated the PM2.5‐induced PWV increase in the carotid artery (*p* < 0.05) (Figure 2B).

**FIGURE 2:**
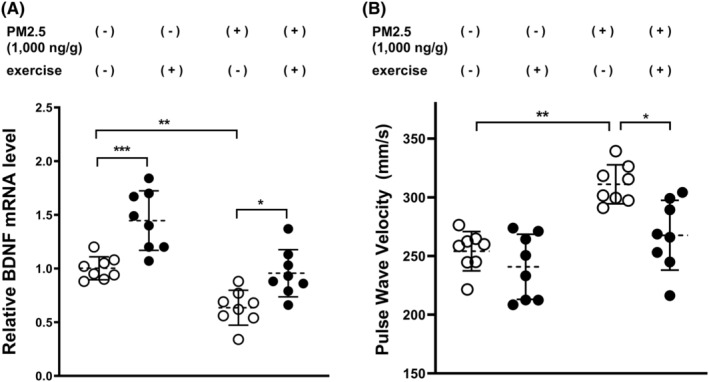
The effect of exercise on endothelial BDNF expression and carotid artery stiffness on Week 26 after PM2.5 dosing at 1000 ng/g for 14 days. (A) The PM2.5‐dosed mice were trained on the treadmill for eight consecutive weeks since the PM2.5 injection started. On Week 26, the exercise was shown to dramatically promote BDNF expression (*p* < 0.01). PM2.5 significantly inhibited BDNF production in the endothelial cells, which could be rescued by exercise. (B) This phenomenon was accompanied by dramatic alleviation of PM2.5‐induced PWV increase in the carotid artery (*p* < 0.05).

Ambient air pollution, especially the PM, has a serious impact on human health. However, it remains unclear what is the mechanism behind PM2.5‐induced stroke. Previous studies indicated that PM2.5 can evade respiratory barriers and enter the circulation,^1^ which then systemically activates leukocytes and inflammatory cytokines.^6^ Since atherosclerosis is known as a chronic inflammatory disease, we hypothesize that PM2.5 can induce carotid artery atherosclerosis. Interestingly, a high dosage of PM2.5 (1000 ng/g) dramatically increased the PWV of the carotid artery starting on Week 18. To our knowledge, this is the first study that has ever identified the carotid artery stiffness after PM2.5 exposure. Additionally, our finding also agreed with the conclusion from the literature, which demonstrated a positive association between PM2.5 and atherosclerotic plaque formation.^6^ Hence, our data confirmed the toxic effect of PM2.5 on the carotid artery and, excitingly, suggested the toxic role of PM2.5 on the cerebral artery and subsequent stroke.

It is well known that BDNF is a key factor in nervous system development as well as normal vascular physiology maintenance.^7^ The endothelial cells are suggested to be the major source of BDNF for CNS, as it has the highest concentration^4^ and production rate^8^ inside the body. Thus, PM2.5‐mediated endothelial cell damage could lead to tremendous BDNF expression inhibition, which facilitates subsequent arterial and neuronal injuries. Our data showed that PM2.5 inhibited BDNF expression in a concentration‐dependent manner in the endothelial cells of the carotid artery. This finding was consistent with the observation of increased arterial PWV, as the loss of BDNF led to decreased vascular protection against PM2.5. A recent study reported that activation of NLRP3 inflammasome and pyroptosis might be the toxicological mechanism of PM2.5,^9^ which also agreed with our results as inhibition of BDNF could interrupt the balance between pro‐ and anti‐inflammatory signals inside the body.

A previous study observed peripheral blood BDNF increase after exercise.^5^ Here, we are the first to report prominent BDNF elevation in the endothelium of the carotid artery after exercise. Excitingly, this elevated BDNF alleviated PM2.5‐induced arterial stiffness. The literature stated that BDNF has a protective effect on endothelium.^7^ Our data agreed with these findings by demonstrating the negative correlation between BDNF and PWV, which indicated that BDNF has a protective effect over the adverse impact of PM2.5 on the carotid artery. Moreover, this conclusion is useful in reducing stroke risk of PM2.5 for human beings living in highly PM2.5‐polluted regions.

Several limitations of our study should be considered. We utilized the intraperitoneal pathway rather than respiratory exposure to achieve a standard experiment condition as previously reported.^10^ We will compare the difference between respiratory and intraperitoneal PM2.5 exposure on the cerebral/carotid artery in future studies. Moreover, PM2.5‐induced artery stiffness is a multifactor phenomenon. Since we only studied the BDNF expression changes inside the endothelial cells after PM2.5 exposure, further study is required to gain a deeper understanding of the mechanism behind this observation.

Conclusively, we demonstrated that PM2.5 could cause carotid artery stiffness by interrupting BDNF expression inside the endothelial cells. Exercise could elevate endothelial BDNF, which alleviates the PM2.5‐induced pathological process in the carotid artery. Thus, we believe that exercise has a beneficial effect on alleviating PM2.5‐induced carotid artery stiffness, which might protect individuals from stroke attacks.

## FUNDING INFORMATION

This work was supported by the Health and Family Planning Project of Pudong Health Committee of Shanghai [No. PW2020E‐2], and Science and Technology Innovation Plan of the Shanghai Science and Technology Commission [No. 201409002500].

## CONFLICT OF INTEREST STATEMENT

The authors declare that they have no conflict of interest.

## Data Availability

The data that support the findings of this study are available from the corresponding author upon reasonable request.
